# Indian scales and inventories

**DOI:** 10.4103/0019-5545.69273

**Published:** 2010-01

**Authors:** S. Venkatesan

**Affiliations:** Department of Clinical Psychology, All India Institute of Speech and Hearing, Mysore - 570 006, Karnataka, India

**Keywords:** Clinimetrics, psychometry, Indian scales and inventories, Diagnostic assessment, Behavior assessment

## Abstract

This conceptual, perspective and review paper on Indian scales and inventories begins with clarification on the historical and contemporary meanings of psychometry before linking itself to the burgeoning field of clinimetrics in their applications to the practice of clinical psychology and psychiatry. Clinimetrics is explained as a changing paradigm in the design, administration, and interpretation of quantitative tests, techniques or procedures applied to measurement of clinical variables, traits and processes. As an illustrative sample, this article assembles a bibliographic survey of about 105 out of 2582 research papers (4.07%) scanned through 51 back dated volumes covering 185 issues related to clinimetry as reviewed across a span of over fifty years (1958-2009) in the Indian Journal of Psychiatry. A content analysis of the contributions across distinct categories of mental measurements is explained before linkages are proposed for future directions along these lines.

## INTRODUCTION

For long, the discipline of ‘psychometry’, as a branch of psychology, dealt with the design, administration, and interpretation of quantitative tests, techniques or procedures for measurement of psychological variables, mental traits and processes like intelligence, aptitude and personality. The term literally means mental measurement. Much earlier, psychometry was viewed as one’s ability to sense or read an object or another person merely by looking, holding or touching. In this historical sense, it was a form of scrying, forecasting or predicting the future – a psychic way of “seeing” something not ordinarily seeable. For example, a psychometrist could hold a watch or ring to tell about the history of that object or the person who owned it-all from what was ‘recorded’ in the object in the form of ‘eminitions’.[1]

Modern psychometry goes beyond such spiritualism, occultism and animistic beliefs. It favors mental measurement as in physical sciences. Scientific psychology re-defined itself severally until it views itself now as ‘science of behavior’. The term ‘behavior’ describes ‘observable and measurable’ actions. In doing so, EL Thorndike declared that ‘anything that exists; exists in some measure’.[2] Measurement is assignment of numerals to objects and events according to some rule. There are reports on an ancient Chinese practice of using mental tests to decide on promotions for civil servants.[3] Much later, Francis Galton, designated father of psychometrics, made efforts to measure intelligence using mental tests. Another tradition of ‘mental measurements’ is connected to psychophysics by Fechner, Weber, W Wundt, JM Cattell and C Spearman. LL Thurstone became the founder-president of Psychometric Society (1936). Spearman and Thurstone developed the theory/application of factor analysis, a statistical method used in psychometrics.[4]

## MODERN PSYCHOMETRY

Psychometrics has now evolved as study on theory and technique of psychological measurements. It includes measurement of knowledge, skills, abilities, aptitudes, attitudes, intelligence, memory, creativity, adjustment and personality. The field uses measuring devices like questionnaires, schedules, rating scales, inventories, checklists and tests. It involves research related to construction of instruments and procedures for measurement, development and refinement of theoretical approaches to mental measurement. More recently, psychometry is being applied to measure beliefs, interests, motivation, academic achievement (reading, writing, spelling or arithmetic) and health-related issues. Measuring these unobservable phenomena is difficult. Attempts are made to define and quantify such phenomena. Some key concepts in psychometrics are: Reliability and validity, training prerequisites of testers, accommodating the notion of individual differences, accepting the inevitable role of measurement errors, considering cross cultural variations, issues related to fairness in testing and test use, rights/ responsibilities of test takers, testing individuals of diverse backgrounds or those under special categories. There are standards related to testing applications, responsibilities of test users, testing in clinical practice, or for program evaluation and public policy. The four basic principles in psychometric testing and test development are reliability, validity, standardization, and bias.[5]

## PSYCHOMETRIC APPROACHES

Traditional psychometry relied on normative approaches to psychological assessment. For example, a person’s level of anxiety is deduced from his score on a test. Then, he is compared reliably with another similar or different population of individuals. Such comparisons with a “norm group” enabled statistically based diagnostic decisions in actual practice. Standardization provided benchmarks for assessing a person against a criterion or norm. In criterion-referenced testing, the scores relate directly to the individual’s competencies. For example, it may be to ascertain whether or not the person has reached a particular standard of expected behavior. Then, there are behavioral assessments which look into contemporary behaviors in individuals and are directly linked to planning/implementing programs for their remediation. In recent times, inspired by neuropsychology, idiometric approaches to assessment is gaining momentum based on a search for underlying common denominators for overt behavioral deficits. For example, ones inability to button, unbutton, pick up small objects, or hold a pencil may all be construed as a common fine motor deficit-which needs to be trained to enable all these or such related activities for that individual.[6–9]

## MERITS AND DEMERITS

Properly developed psychometric tools, when used by competent and qualified individuals, have several advantages. They are relatively cheap, easy to administer, or lead to valid judgments. To put it simply, information from a good personality questionnaire, for example, might take several months of knowing and working with a person. They are likely to be of considerable cost-benefits in the long term. Whether to aid a clinical diagnosis or plan an intervention program, the expenses involved in psychometric testing is minimal compared to the costs of high-turn over, under-performance or misdiagnosis of clients. There are also perils in psychometric testing. Many tests and questionnaires are let into the market in the guise of ‘psychometric instruments’. It is difficult to distinguish ‘genuine’ ones. These instruments are put together by people with no background in psychometrics. They have little actual utility and value for the purposes for which they are marketed. There is also a danger of their use by untrained examiners even though there is indeed, no guarantee that even trained person will at all times use them correctly. In short, psychometric tests and procedures are useful but are not necessarily universal solutions.[10]

## APPLICATIONS

Psychometry has wide applications in business, industry, recruitment, personnel selection, executive coaching, teambuilding, management development and job placement, educational testing, selection, deciphering learning styles, enabling career choices, profiling individual or group behavioral assets and deficits. In recent times, it is being applied to the field of health, illness, disease, and disability behaviors. As applied to clinical medicine-especially psychiatry, psychometric tests can be viewed in two broad categories: Knowledge based and person based.[11] Tests of ability, aptitude, attainment, competence, and achievement are examples of the former, while tests of personality, clinical symptoms, mood, integrity, interests, and attitude typify the latter. Knowledge- based tests have right-wrong answers. Person-based tests differentiate different types of individuals. Within knowledge-based testing, for example, tests of clerical aptitude, computing skills, and numerical and verbal reasoning have relevance to different types of work. Speech and language therapists use tests that assess dyslexia, language impairment, and other forms of educational underachievement. Neuropsychological tests cover cognitive functions like memory and abstract thinking. Personality tests in clinical practice decipher pre- or post morbid changes in habitual, temperamental or regular patterns of behavior in an individual. A problem for all personality tests is ‘lying’ or ‘faking good’. Much effort is put into test construction to circumvent this tendency. Today, psychometric testing has the widest applications. Most of us can expect to be tested at least once in our working lives. Because psychometrics is such a powerful tool, it is essential that it is applied responsibly.[2]

## PSYCHOMETRY TO CLINIMETRY

For long, assessment in psychiatry or clinical psychology did not cover quantification. Acute but irreproducible long descriptions were used to communicate clinical observations.[12] More objective ways of clinical assessment about the severity and change in symptoms emerged later. Since then, clinical assessments placed stress on inter- clinician reliability. The assessment of clinical changes, the recent trust on psychological instruments which are valid and reliable is at the heart of clinimetrics.[13] In its quest for valid and reliable assessment, clinimetry rest its foundations on rather the clinically shaky grounds of psychometric theory. Note that developments in classical and modern psychometry took place outside of the clinical field-mainly in educational and social areas. Hence, they could not be automatically adjusted to clinical fields.[14] The inadequacies of the psychometric model in clinical setting have spurred the need for supplementation with another conceptual framework: clinimetrics!

### Inadequacy of psychometry model

The inadequacies of psychometrics in clinical setting were first identified by Shapiro in 1951 and described in relation to assessment of changes in distress.[15] Sensitivity to behavior change is a requisite for clinical validity of an outcome scale. Scales may be valid and reliable. But, they may lack sensitivity.[16] This concept is important when treatment effects are small and in the setting of sub-clinical symptoms.[17,18] The psychometric model is inadequate in clinical setting owing to its search for homogeneity. Homogeneity of components, as measured by statistical tests such as Cronbach’s alpha, is often seen as the most important requirement for a traditional rating scale. However, the same properties that give a scale a high score for homogeneity may obscure its ability to detect change.[19] The redundant items in a scale may increase Cronbach’s alpha, but decrease its sensitivity. In psychometrics, a high correlation is often regarded as evidence that the two scales measure the same factor. However, a high correlation does not indicate similar sensitivity. When a new scale is developed with “item analysis”, some of the essential variables that are sensitive to change may be either removed or not included.[18]

Several clinical scales, such as the Hamilton Depression Scale[20] was developed based on classical psychometric model. A key flaw of such instruments, developed on the basis of factor analysis or principle component analysis (in which correlation coefficients are operating by giving symptoms equal weights), is that the same score at this Scale may be the product of few very severe core symptoms (example a severely retarded depressed patient) or of several mild accessory symptoms (reflecting perhaps a subject affected by a mild form but with many symptoms and a complaining behavior)[21] An alternative model, clinimetrics is being increasingly proposed as the conceptual basis to assess clinical phenomena, diagnosis, prognosis, and therapeutics.[22,23]

### Clinimetrics

The term “clinimetrics” was introduced by Alvan R. Feinstein in 1982 to indicate a domain concerned with indexes, rating scales and other expressions that are used to describe or measure symptoms, physical signs, and other distinctly clinical phenomena. The purpose of clinimetrics is to provide an intellectual home for a number of clinical phenomena. It includes the types, severity and sequence of symptoms; rate of progression of illness, severity of co-morbidity; problems of functional capacity; reasons for medical decisions; and many other aspects of daily life, such as well-being and distress.[24] A familiar example of clinimetric index is Apgar’s method of scoring the newborn’s condition.[25] Clinimetrics has a set of rules which govern the structure of indexes, the choice of component variables, the evaluation of consistency and validity.[26]

Clinimetrics has an advantage over classical psychometric measures in being more sensitive to symptom change. It uses a sensible method to assess symptoms based on their prevalence in those with a disorder (clinical coherence) and the importance of those symptoms for clinicians to define severity (weighting of symptoms).[22] The concept of incremental validity refers to the unique contribution or incremental increase in predictive power associated with the inclusion of a particular assessment procedure in clinical decision process.[27] A clinimetric, instead of psychometric model should guide the diagnosis in psychiatry and clinical psychology.

Clinimetrics stresses multiple points of observation during the course of illnesses by calling in fact for a substantial modification of the flat, cross-sectional approach based on singular official criteria only. A longitudinal consideration of the development of disorders may prove to be more fruitful for clinical decision making and treatment planning than a cross-sectional diagnosis.[12,17,28] An example of this approach is a recent publication on the historical analysis on course and clinical presentation of children with learning disabilities in India,[29] wherein it was demonstrated that the signs and symptoms, presentation or clinical course of the condition range from specific speech delays during preschool ages, to difficulties in pre-academic activities and writing problems during kindergarten ages, followed by mixed disturbances in reading, writing and spelling problems during early primary school years. This is followed by reported clinical presentations of behavioral difficulties during middle school ages of these children. The condition gets further disguised as problem behaviors and present either externalized as conduct disorder or internalized into emotional disturbances. Much later, during pre-adolescence or adolescence, the learning disability manifest either as severe neurotic disturbances like depression, anxiety, obsessions, compulsions and phobias or as prodromal symptoms of newly shaping passive aggressive, antisocial, or some such personality disorders.

Clinimetrics is in accordance with sequential model of treatment, which was found to be effective in psychiatry. It has clinical implications in the definition of recovery. Commonly, recovery reflects ‘improvement’ or the clinical distance along which the current state of the patient is compared to the pre-treatment position.[30] In this sense, recovery is expressed as categorical variable (present/absent) or as comparative category (non-recovered, slightly recovered, moderately recovered, greatly recovered). Both expressions require arbitrary cut-off points related to amount of improvement. Clinimetrics offers conceptual and methodological ground for a substantial revision of assessment parameters and for linking co-occurring syndromes. By a research viewpoint, it may pave the way for inclusion criteria and assessment tools which are more suitable for the purposes of clinical research. Rigid adherence to the psychometric model may only prevent such progress in clinical testing.

## SCENE IN INDIA

Psychometry applied to psychiatry/clinical psychology (or clinimetrics) is of a recent origin in the country. These parent disciplines are themselves still struggling to establish themselves as distinct health delivery systems against the backdrop of the slush in laymen preoccupation or first preferences for magic-religious traditional treatments for psychologically affected individuals or their families. Combine this with the grim situation of growing rural-urban or rich-poor divide, gaping illiteracy, multiplicity of castes and sub-cultures, linguistic plurality, religious jingoism and gender differences in the larger populations-all of which contribute to the scenario of poor understanding of westernized paper pencil tests, or acceptance of psychometry based clinical testing. Despite these limitations and challenges, the field of clinimetrics has witnessed a periodic although unsteady or patchy growth in mental health practice in the country. Such research developments have been intermittently published in some applied psychology and psychiatry journals. In the following, publications related to clinimetry in Indian Journal of Psychiatry (IJP) between October-December, 1958 and July-September, 2009 are summarized and discussed.

## CONTRIBUTIONS BY INDIAN JOURNAL OF PSYCHIATRY

The IJP is an official quarterly publication of Indian Psychiatric Society. It publishes peer-reviewed original work related to psychiatry. For purpose of this review, 51 back dated volumes covering 185 issues (excluding supplements, editorials, guest editorials, letters to editor, presidential addresses, book reviews, award papers, or reports, theoretical reviews, etc) were individually scanned for a complete and comprehensive bibliographic search on original articles, research papers and submissions related to clinimetrics. The term ‘clinimetric’ as operationally defined in this paper refers to any or all those research articles which are directly worked on clinical populations related to test construction, validation, field-try out, standardization, application and/or their translations. A complete review of clinimetric contributions in IJP reveled 105 research articles in about 2582 research papers (4.07 %) surveyed across a span of over fifty years (1958-2009) [Table 1]. A further content analysis of such clinimetric contributions in the journal showed that the reviewed scientific papers broadly fell under four distinct categories of mental measurements:

**Table 1:** Distribution of research articles on clinimetric measurements in Indian journal of psychiatry (1958-2009)

Year/s	Total	Personality	Cognitive/organic	Diagnostic	General	Total
<1970	339	6	1	1	2	10
1971-1980	521	14	3	10	3	30
1981-1990	705	2	2	16	2	22
1991-2000	546	1	2	5	21	29
2001>	471	1	5	3	5	14
TOTAL	2582	24	13	35	33	105

### Personality

Research articles pertaining to personality measures and studies have been amongst one of the earliest concerns in modern Indian psychiatry as evidenced by the use of projective techniques, such as, Rorschach Inkblots for various clinical groups including schizophrenics,[31,32] murderers,[33] or other psychiatric cases.[34] Another projective technique, namely Draw a Person Test was also tried on homosexuals in another related study.[35] Simultaneously, early research interest was on development/use of other personality measures like inventories,[36] especially Eysenck,[37,38] Maudsley,[39,40] MMPI[41] or in mutual comparisons with one another.[42] Later, there was a brief interest in getting authenticated translated versions of these inventories in vernacular[43–46] or studying specific sub aspects of personality types or profiles in relation to specific psychiatric manifestations.[47–49] In this connection, measurement of personality traits-either psychoticism-neuroticism,[50] authoritarianism[51] or in consonance with particular psychiatric disturbances was some of the beginning concerns in this field.[52,53] During the period surveyed in this study, there were in only 6 inventories studied by contributors to the IJP in the area of personality in psychiatry [Table 2].

**Table 2:** Distribution of scales and inventories worked upon in Indian journal of psychiatry (1958-2009)

Title of scale/inventory	Area/domain tested
Rorschach ink blot technique	Personality
Draw a person test	Personality
Eysenck personality inventory	Personality
Maudsley personality questionnaire	Personality
Minnesota multiphasic personality inventory	Personality
Bortner rating scale for type A Personality	personality
Luria nebraska neuropsychological test battery	Cognitive
Wisconsin card sorting test	Cognitive
Bender gestalt test	Cognitive
PGI memory scale	Cognitive
Cattell’s infant intelligence scale	Cognitive
Middlesex hospital questionnaire	Diagnostic
PGI health questionnaire	Diagnostic
Beck depression inventory	Diagnostic
General health questionnaire	Diagnostic
Depression-Happiness scale	Diagnostic
Hamilton depression scale	Diagnostic
Goldberg general health questionnaire	Diagnostic
16 PF questionnaire	Diagnostic
Suicidal intent questionnaire	Diagnostic
Illness behavior questionnaire	Diagnostic
Scale for assessment of negative symptoms in schizophrenia	Diagnostic
Childhood Psychopathology measurement schedule	Diagnostic
Motivation scale for alcohol dependence	Diagnostic
Alcohol dependence data questionnaire	Diagnostic
Brief psychiatric rating scale	Diagnostic
Composite international diagnostic interview	Diagnostic
Family interaction patterns scale	Social
Socio-Economic status scale	Social
Parental handling questionnaire	Social
Scalef or assessment of psychiatric disability	Social
Maternal attitude and maternal adjustment scale	Social
Sex knowledge and attitude questionnaire	Social
Sensation seeking scale	Social
Brief addiction rating scale	Social
SCARF social functioning index	Social
Burden assessment schedule	Social
Stressful life events and social supports inventory	Social
Quality of life interview	Social

### Cognition

In the area of measurement of cognition, various aspects have caught the early interest of researchers. The ‘Bender Gestalt Test; was tried to discriminate organic/functional disturbances in psychiatric patients.[54–56] Later, the study of thought disturbances in major mental disorders was focused,[57] along with difficulties in recognition,[58] and memory.[59] Almost after a decade and half later, interest in cognitive research underlying mental disorders got revived with the belated arrival of neuropsychology as a discipline.[60–63] Although not adequately knit, these interests are continuing sporadically and disjointedly until recently.[64–66] During the period surveyed, there were in all 5 tests worked upon by contributors in the area of cognition [Table 2].

### Diagnostic

Diagnostic psychiatry has always waited for psychometric tools to be the look alike of pathology tests, to aid in screening, identification of quick diagnosis of various clinical conditions. It has always wanted an aide to objectively and efficiently help in diagnostic decision making. In this pursuit, after the era on projective techniques, one can distinctly see several paper pencil tests or clinical scales emerging in their place, such as, Middlesex Hospital Questionnaire,[67–70] PGI Health Questionnaire,[71,72] Beck Depression Inventory,[73] General Health Questionnaire,[74–76] Goldberg General Health Questionnaire,[77] Depression-Happiness Scale,[78] Hamilton Depression Scale.[79] The purpose and expectation from these tests/measures was to tell the diagnostician whether a given patient had a particular disorder or not based on a numerical score or cut off point. No wonder, this expectation from psychometric tests was to parallel pathology tests in medicine to enable the physician to decide whether a patient has a particular disease is reflected in the highest number of 16 such instruments researched by contributors to IJP [Table 2].

### Others

In recent times, clinimetrics as applied to Indian psychiatry appears to be moving away from its rather rigid medical model. It is beginning to appreciate the importance of attitudes, opinions, and similar other soft skills in patient care/ improvement. This is evidenced by studies published in IJP related to families, their perceptions,[80] interaction patterns,[81] illness behavior,[82,83] social functioning,[84] burden,[85,86] coping strategies,[87,88] handling patterns,[89] social supports,[90] attitudinal variations,[91–95] stressful life events,[96–98] quality of life,[99,100] etc. These recent concerns on psychometric instruments related to social dimensions of psychiatry are reflected as research works on 12 scales by contributor to this journal [Table 2]. In terms of life span perspective, there was one paper on clinical psychometry applied to pediatric psychiatry of infantile autism,[101] another on infant intelligence scales,[102] childhood psychopathology[103] or their temperamental characteristics.[104] There were no scales related to adolescent psychiatry and there was one paper on scale for old age psychiatry.[105]

## CRITICAL REVIEW

From the foregoing, it is evident that clinimetrics has received low priority in the pages of IJP over the years. There are many areas, problems and issues in contemporary mental health practice wherein inter-disciplinary/multi-disciplinary inputs are needed. An earlier lament for greater objectivity in psychiatric research is true even today.[106] One can wish away this shortcoming by defending that test development appropriately falls in the domain of a sibling discipline in clinical psychology; or that psycho-social issues in psychiatry research vests with psychiatric social work. But, this defense is untenable if one wishes to rise above biological psychiatry into community/social psychiatry, industrial psychiatry, preventive mental health, school mental health, etc. With a new breed of changed social scenario of ready made two- minute mendicants and missionaries preaching spiritual peace or tranquility and what with increasing life style disorders, transitional difficulties, culture bound recessions, changed systems of education, IT enabled and materialistic living bereft of genuine spiritual musings, the contemporary social scene is fertile for development of psychopathology.[107,108]

The review shows neglect on mental health issues related to old age and elderly, women and domestic violence, transgender, and disabled. Take the example of an extensive bibliographical research in the field of mental retardation undertaken by Indian investigators on populations within the country and published in over 100 national journals spanning sixty years.[109] It was found that in the surveyed period, there were no more than 1095 (68.91%) research articles on mental retardation, 15 (0.94%) papers on autism, and only 4 papers (0.24%) on learning disabilities. Among many other findings, it was seen that the IJP stood at seventh rank (3.84%) after ‘Indian Journal of Pediatrics’ (19.63%) and ‘Indian Pediatrics’ (12.15%) in terms of number of publications on mental retardation. Apart from commenting on the quality or repetitiveness of studied areas in the undertaken research, this study concluded that disability is the most neglected area of study by all professionals. There were hardly tools/scales on early intervention, prevention, inclusive education, consumer behaviors, professional conduct, changing perspectives/definitions of disability, mainstreaming, community based initiatives, access audit, empowerment issues, community impact evaluations, historical analysis, cost-benefit studies, etc. Clinimetric devices need to be developed and standardized in several of these unaddressed areas.

Another line of needed research is periodic revalidation of antique tests. Ours is probably the only country where testers continue to use decade old versions of tests, inventories and/ or scales to make standard comparisons of individuals in the present ‘super computer and information age’ with norms and manuals prepared in the west or those prepared in ‘before-man-in-the-moon’ era of our country. In most instances, there are no adaptable or adjustable norms for mentally ill, those with special needs, minority groups, the rural, under privileged, neglected, discriminated and marginalized. Though one may fault normative approaches and attempted comparisons between individuals, for purpose of certification and tentative impressions on diagnosis, periodic up grading of such norms is indispensable. Therefore, cues may be taken from papers addressed to reporting revalidated norms for popular tests, such as, Bender Gestalt Visuo Motor Test,[110] Binet Kamat Intelligence Scale,[111] Gessell Drawing Test,[112] Seguin Form Board,[113,114] Play Activity Checklist for Mental Retardation,[115] Mathematics Anxiety Rating Scale,[116] Parental Attitude Scale[117] etc.

Diagnostic testing and statement of deviations or delay in a given individual from the so called ‘normal’ does not complete the job of a clinimetrician. In fact, this is just the beginning of identification for certification. However, the real work would be to plan intervention programs. There is a need for structured, systematic and standardized intervention packages for persons with mental ill health problems. Such packages need to be comprehensive, flexible, field tested, viable, functional, objective, observable and measurable-all and at the same time Indian at heart. An attempted answer for this problem was the development and standardization of ‘Behavior Assessment Scales for Children with Mental Retardation’,[118] the ‘Activity Checklist for Preschool Children with Developmental Disabilities’[119] now the ‘Assessment of Kids with Special Handicaps in Arithmetic and Reading-Writing Activities’.[120] More such intervention based ready-made/easy-to-use Indian scales are required to meet exclusive needs of kids with multiple handicaps, severe-profoundly affected individuals and/or their families. And, what’s more! This entire package must come in regional languages. This is the understanding and logic behind the now popular ‘Toy Kit for Kids with Developmental Disabilities’[121] made available in English and Kannada. Things are definitely changing. Take the instance of the claimed phenomenon of increase in numbers of autism and learning disabilities (a terrible term word for kids with average intelligence and a cruel curriculum imposed on them). Clinical psychometry has a far greater role and responsibility towards several thousands of such children out there in every school-more than simply issuing a certificate or report of learning disability!

Formal testing devices are needed to explore consumer demand for services in the area of mental health, problems and issues related to management of mentally ill persons in home settings. There are differential self and other perceptions on or about affected individuals, their caregivers, siblings and family which need to be explored in the local context. In this era of information age, contemporary clinimetrics needs to re-adapt, shed ancient attitudes and ways of testing, blend with available gadgetry. A sample of this kind is ongoing work on development and standardization of a software program and expert system to enable computerized testing, diagnostic decision making apart from intervention planning and programming for individual children with developmental disabilities.[122]

Online assessments, chat rooms, e-based discussions, consultations and therapeutic self help groups are becoming increasingly popular even in our country. A recent paper on content analysis of transcripts derived from data mining of 3436 email exchanges in a organized internet group on of netizens revealed that many parents are lost in the quagmire of information overload as they discuss/seek more than 238 types of treatment for children on the autism spectrum.[123] Unless the contemporary practitioners become computer savvy, there is likelihood of their being left behind in the ongoing race between man and machine. Further, the calamitous outcome of over involvement of contemporary human living with machines in preference for human interactions have also resulted in loss of social niceties, emotive skills, person to person exchange competencies, and the like-all of which is an important material for investigation in the field of positive mental health. This has been demonstrated in another paper on the 24- hour activity log of typical kids on the autism spectrum and those with developmental disabilities which reported the amount of time spent per day on needed constructive activities like ‘home teaching’ (4.32%) or ‘playing with peers’ (4.12%) are meager.[124] It is one thing to innovate, create, design or develop models or services for special populations of individuals in the country. It is quite another thing to disseminate, distribute and dispense them for the ultimate benefit of end users.

## EPILOGUE

There are a wide range of psychometric tests as there are psychometric examiners-many of who may be untrained, inexperienced and confused too. The application of psychometrics to psychiatric practice is called clinimetrics. Clinimetric tests are useful for collecting huge mass of data or for certain routine clinical tasks. The test findings must be viewed only as a kind of random sampling of the individual/s behavior obtained under controlled conditions in terms of their responses to questions and reactions to various situations. The score of an adequately standardized test must be only taken as an indicative index of the psychological variable being measured. This field may be viewed as analogous to that of laboratory technicians in the field of medicine. In dynamic clinical work, traditional psychometry would be just as effective as the technicians report in the hands of a physician. No physician would end up making a diagnosis based on a laboratory report alone. Similarly, no psychiatric diagnostician must make or take a decision based on psychometric findings alone to the exclusion of other adjunct methods like case history, direct clinical observation, individual or family interview, mental status examination, family history, etc.

## References

[1] Jessup JW (2004). The Yeshua Codex.

[2] Thorndike RL (1982). Applied Psychometrics.

[3] Wainer H (2000). Computer Adaptive Testing: A Primer.

[4] Nunnally J, Bernstein I (1994). Psychometric Theory.

[5] Anastasi A, Urbina S (1997). Psychological Testing.

[6] Venkatesan S, Reddy KR (1990). Idiometric approaches to functional assessment in mentally handicapped adults: Report of a pilot study. Indian J Clin Psychol.

[7] Venkatesan S, Reddy KR (1991). Validation of an idiometrically based functional assessment battery for mentally handicapped adults. Indian J Clinical Psychol.

[8] Venkatesan S, Reddy KR (1993). Sensitivity of an idiometrically based functional assessment battery to changes in neuropsychological functions in adults with mental handicap. Creative Psychol.

[9] Venkatesan S (1994). Recent trends and issues in behavioral assessment of individuals with mental handicap in india. Creative Psychol.

[10] Guilford JP (1936). Psychometric methods.

[11] Rust J, Golombok S (1999). Modern Psychometrics: The science of psychological assessment.

[12] Fava GA, Belaise C (2005). Clinical assessment: The role of clinimetrics and the misleading effects of psychometric theory. J Clin Epidemiol.

[13] De Wet HC, Terwee CB, Bouter LM (2003). Clinimetric versus psychometrics: Two sides of the same coin. J Clin Epidemiol.

[14] De Wet HC, Terwee CB, Bouter LM (2003). Current challenges in clinimetrics. J Clin Epidemiol.

[15] Feinstein AR (1983). An additional science for clinical medicine. IV. The development of clinimetrics. Ann Intern Med.

[16] Streiner DL (2003). Clinimetrics vs psychometrics: An unnecessary distinction. J Clin Epidemiol.

[17] Fava GA, Ruini C, Rafanelli C (2004). Psychometric theory is an obstacle to the progress of clinical research. Psychother Psychosom.

[18] Fava GA (2006). The Intellectual Crisis of Psychiatric Research. Psychother Psychosom.

[19] Wright JG, Feinstein AR (1992). A comparative contrast of clinimetric and psychometric methods for constructing indexes and rating scales. J Clin Epidemiol.

[20] Hamilton M (1967). Development of a rating scale for primary depressive illness. Br J Soc Clin Psychol.

[21] Faravelli C (2004). Assessment of psychopathology. Psychother Psychosom.

[22] Bech P (2004). Modern psychometrics in clinimetrics: Impact on clinical trials of antidepressants. Psychother Psychosom.

[23] Nierenberg AA, Sonino N (2004). From clinical observations to clinimetrics: Atribute to Alvan R. Feinstein. Psychother Psychosom.

[24] Feinstein AR (1982). The Jones criteria and the challenge of clinimetrics. Circulation.

[25] Feinstein AR (1999). Multi-item “instruments” versus Virginia Apgar’s principles of clinimetrics. Arch Intern Med.

[26] Feinstein AR (1987). Clinimetrics.

[27] Wimmer A (2003). Psychogenic psychosis. 1st ed in Danish 1916.

[28] Feinstein AR (1990). The inadequacy of binary models for the clinical reality of three-zone diagnostic decisions. J Clin Epidemiol.

[29] Venkatesan S, Purusotham P (2006). Historical analysis on course and clinical presentations of children with learning disabilities in india. Disabil Impair.

[30] Savron G, Fava GA, Grandi S, Rafanelli C, Raffi AR, Belluardo P (1996). Hypochondriacal fears and beliefs in obsessive-compulsive disorder. Acta Psychiat Scand.

[31] Khorana S (1970). Pattern of rorschach responses in schizophrenic and normal male children. Indian J Psychiatry.

[32] Bagadia VN, Anand S, Saraf KR, Shah LP (1971). Analysis of rorschach test of 250 cases of schizophrenia. Indian J Psychiatry.

[33] Sethi BB, Gupta SC, Raj AS, Nathawat SS (1971). Rorschach as a measure of psychopathology in murder. Indian J Psychiatry.

[34] Ardhapurkar IB, Doongaji DR (1965). A Selective study of rorschach records-209 cases. Indian J Psychiatry.

[35] Sreedhar KP, Rao VA (1973). Draw A person test in two male homosexuals. Indian J Psychiatry.

[36] Gupta SC (1966). A Personality Inventory. Indian J Psychiatry.

[37] Verghese A (1969). Eysenck personality n scores in different diseases. Indian J Psychiatry.

[38] Verghese A, Sundar P, Rao PSS, Abraham A (1971). The E.P.I. scores in a group of candidates for admission into a medical college. Indian J Psychiatry.

[39] Jain IS, Prasad M, Gupta SC, Singh K (1972). Evaluation of the personality of the blind through maudsley personality inventory. Indian J Psychiatry.

[40] Sreedhar CP, Chinnian RR, Rao VA (1974). The Maudsley personality inventory scores in a group of psychiatric patients. Indian J Psychiatry.

[41] Deshmukh DK, Eswaran S, Nagesh RP, Mahajan S (1980). A comparative study of MMPI Profiles of Psychiatric Pgs V/s Medical Pgs. Indian J Psychiatry.

[42] Gupta AK, Sethi BB, Gupta SC (1976). EPI and 16 PF Observations in smokers. Indian J Psychiatry.

[43] Abraham A, Rao PS, Verghese A (1977). Standardization of vernacular translations of the eysenck personality inventory. Indian J Psychiatry.

[44] Verma SK, Wig NN (1977). Standardization of a neuroticism questionnaire in hindi. Indian J Psychiatry.

[45] Arora M, Varma VK (1980). A Psychoticism Scale in Hindi: II Standardization. Indian J Psychiatry.

[46] Arora M, Varma VK (1980). A Psychoticism scale in hindi: I. Construction and initial tryouts. Indian J Psychiatry.

[47] Raju GG, Krishnaswamy S, Verghese A, Mahadevappa H (1987). Usefulness of bortner rating scale in the measurement of type A behavior pattern. Indian J Psychiatry.

[48] Sahasi G, Chawla HM, Bhushan B, Kacker C (1990). Eysencks personality questionnaire scores of heroin addicts in india. Indian J Psychiatry.

[49] Kannapiran T, Haroon AE, Vivekanandan S, Arnagiri S (1997). Personality profiles of self immolators. Indian J Psychiatry.

[50] Pershad D, Verma SK, Wig NN, Wahi PL (1972). Measures of neuroticism and prediction of psychiatric disturbances in patients with cardiac surgery. Indian J Psychiatry.

[51] Varma VK, Akhtar S, Kaushal P, Vasudeva P, Kulhara PN (1973). Measurement of authoritarian traits in india. Indian J Psychiatry.

[52] Abraham A, Verghese A (1973). The Association between EPI scores and academic achievement among medical students. Indian J Psychiatry.

[53] Wig NN, Pershad D, Verma SK, Menon DK (1977). Usefulness of certain personality tests for prediction of psychiatric disturbances follow in tubal ligation. Indian J Psychiatry.

[54] Dube KS (1965). The Bender Gestalt Test. Indian J Psychiatry.

[55] Bagadia VN, Doshi J, Pradhan PV, Shah IP (1975). A study of the bender gestalt test in 222 adult psychiatric cases. Indian J Psychiatry.

[56] Pershad D, Verma SK, Malhotra S, Prabhakar S (1984). Screening of organic brain dysfunction. Indian J Psychiatry.

[57] Singh MV (1971). A psychometric approach to schizophrenic thought disorders. Indian J Psychiatry.

[58] Pershad D, Wig NN (1976). A recognition test for clinical use. Indian J Psychiatry.

[59] Pershad D, Wig NN (1978). Reliability and validity of a new battery of memory tests. Indian J Psychiatry.

[60] Nizamie A, Nizamie SH, Shukla TR (1992). Performance on Luria-Nebraska neuropsychological battery in schizophrenic patients. Indian J Psychiatry.

[61] James MX, Nizamie A, Nizamie SH (1997). Comparative study of clinical effectiveness of luria nebraska neuropsychological battery, EEG and CT scan in brain damaged patients. Indian J Psychiatry.

[62] Mishra BP, Gupta V, Mahajan R, Narang RL (2002). Pattern performance of schizophrenic patients on Luria-Nebraska Neuro-psychological battery. Indian J Psychiatry.

[63] Mishra BP, Mahajan R, Dhanuka A, Narang RL (2002). Neuro-psychological profile of epilepsy on luria-nebraska neuropsychological battery. Indian J Psychiatry.

[64] Andrade C, Madhavan AP, Kishore ML (2001). Testing Logical memory using a complex passage: Development and standardization of new test. Indian J Psychiatry.

[65] Kohli A, Kaur M (2006). Wisconsin Card Sorting Test: Normative data and experience. Indian J Psychiatry.

[66] Vahia VN, Bhojraj T, Creado DA (2006). Neuro-cognitive deficits in HIV-positive patients-two case reports: Revising current AANTF, guidelines in view of recent revelation of new neuro cognitive symptoms. Indian J Psychiatry.

[67] Prabhu GG (1972). Clinical utility of The Middlesex Hospital Questionnaire in India. Indian J Psychiatry.

[68] Srivastava ON, Bhat VK (1974). The Middlesex Hospital Questionnaire (MHQ) standardization on a hindi version. Indian J Psychiatry.

[69] Somasundaram O, Mathurubootham N (1979). Standardization of tamil version of Middlesex Hospital Questionnaire. Indian J Psychiatry.

[70] Gada MT (1981). Standardization of gujarati version of Middlesex Hospital Questionnaire. Indian J Psychiatry.

[71] Wig NN, Verma SK (1973). PGI Health Questionnaire N-1: A Simple Neuroticism Scale in India. Indian J Psychiatry.

[72] Wig NN, Verma SK (1993). PGI Health Questionnaire-a simple neuroticism scale in india. Indian J Psychiatry.

[73] Ajmany S, Nandi DN (1973). Adaptation of AT. Beck*et al.*’s An inventory for measuring depression. Indian J Psychiatry.

[74] Shamasunder C, Sriram TG, Muraliraj SG, Shanmugham V (1986). Validity of a short 5-item version of the General Health Questionnaire (GHQ). Indian J Psychiatry.

[75] Jacob KS, Bhugra D, Mann AH (1997). General Health Questionnaire-12: Psychiatric properties and factor structure among indian women living in the united kingdom. Indian J Psychiatry.

[76] Pothen AK, Philip K, Braganza D, Joseph A, Jacob KS (1999). The validation of the tamil version of the 12 item General Health Questionnaire. Indian J Psychiatry.

[77] Gautam S, Nijhawan M, Kamal P (1987). Standardization of hindi version of Goldbergs general Health Questionnaire. Indian J Psychiatry.

[78] Kishore TM, Pal SE (2003). The depression-happiness scale and quality of life in patients with remitted depression. Indian J Psychiatry.

[79] Prasad MK, Udupa K, Kishore KR, Thirthalli J, Sathyaprabha TN, Gangadhar BN (2009). Inter-rater reliability of hamilton depression rating scale using video-recorded interviews-focus on rater-blinding. Indian J Psychiatry.

[80] Gopinath PS, Chaturvedi SK (1986). Measurement of Distressful Psychotic Symptoms Perceived by the Family: Preliminary Findings. Indian J Psychiatry.

[81] Bhatti R, Ageira B (1986). Validation of Family Interaction Patterns Scale Indian J Psychiatry.

[82] Varma VK, Malhotra A, Chaturvedi SK (1986). Illness Behavior Questionnaire (IBQ): Translation And Adaptation In India. Indian J Psychiatry.

[83] Chaturvedi SK, Bhandari S, Rao S (1988). Illness behavior assessment of psychiatric patients with somatic presentation. Indian J Psychiatry.

[84] Padmavati R, Thara R, Srinivasan L, Kumar S (1995). SCARF Social Functioning Index. Indian J Psychiatry.

[85] Roychaudhuri J, Modal D, Boral A, Bhattacharya D (1995). Family burden among long term psychiatric patients. Indian J Psychiatry.

[86] Thara R, Padmavathi R, Kumar S, Srinivasan L (1998). Burden assessment schedule: Instrument to assess burden of caregivers of chronic mentally III. Indian J Psychiatry.

[87] Rao K, Subbakrishna DK, Prabhu GG (1989). Development of a coping checklist-a preliminary report. Indian J Psychiatry.

[88] Chandrasekharan R, Chitralekha V (1998). Patterns and determinants of coping behavior of wives of alcoholics. Indian J Psychiatry.

[89] Malhotra S (1990). A Parental Handling Questionnaire. Indian J Psychiatry.

[90] Kulhara P, Avasthi A, Gupta N, Das MK, Nehra R, Rao AS (1998). Life Events and Social Supports in Married Schizophrenics. Indian J Psychiatry.

[91] Das K, Kulhara P (1991). Maternal Attitude and Maternal Adjustment: Development of a Scale for use in India setting. Indian J Psychiatry.

[92] Avasthi AK, Varma VK, Nehra R, Das K (1992). Construction and standardization of a sex knowledge and attitude questionnaire (SKAQ) in simple hindi, for north indian population. Indian J Psychiatry.

[93] Basu D, Malhotra A, Varma VK, Malhotra R (1998). Development of scale to assess attitudes towards drinking and alcoholism. Indian J Psychiatry.

[94] Satija YK, Advani GB, Nathawat SS (1998). Influence of Stressful Life Events and Coping Strategies in Depression Indian J Psychiatry.

[95] Venugopal D, Ranjith G, Issac MK (2000). A questionnaire survey of psychiatrists attitudes towards genetic counseling. Indian J Psychiatry.

[96] Chandrashekar CR, Reddy V, Issac MK (1997). Life events and somatoform disorders. Indian J Psychiatry.

[97] Kumar PN, Das J, Bagchi DJ, Pal HR (1998). A comparative study of life events in depression and mania. Indian J Psychiatry.

[98] Raju MS, Srivastava K, Chaudhury S, Salujha SK (2001). Quantification of stressful life events in service personnel. Indian J Psychiatry.

[99] Lobana A, Mattoo SK, Basu D, Gupta N (2002). Convergent validity of quality of life interview (QOLI) in indian setting: Preliminary findings. Indian J Psychiatry.

[100] Chaudhury S, Srivastava K, Kamaraju MS, Salujha SK (2006). A Life Events Scale for Armed Forces Personnel. Indian J Psychiatry.

[101] Sreedhar KP, Rao VA (1973). Draw a person test in two male homosexuals. Indian J Psychiatry.

[102] Koshy V, Sharma SD (1984). Standardization of the cattells infant intelligence scale in india. Indian J Psychiatry.

[103] Malhotra S, Varma VK, Malhotra A (1988). Childhood psychopathology measurement schedule: Development and standardization. Indian J Psychiatry.

[104] Narang RL, Gupta R, Mishra BP, Mahajan R (1997). Temperamental characteristics and psychopathology among children of alcoholics. Indian J Psychiatry.

[105] Rao VA (1999). Assessment scales in old age. Indian J Psychiatry.

[106] Prabhu GG (1967). Objectivity in psychiatric research-A review. Indian J Psychiatry.

[107] Venkatesan S (1991). Psychological assessment of individuals with mental retardation: Some perspectives and problems. Creative Psychol.

[108] Venkatesan S, Choudhury S (1995). Psychodiagnostic assessment of rural children with mental handicaps in india: Some problems and issues. Creative Psychol.

[109] Venkatesan S, Vepuri VG (1995). Mental retardation in india: A bibliography.

[110] Venkatesan S (1991). Bender gestalt visuo motor test as measure of intelligence in mentally handicapped individuals. Indian J Clin Psychol.

[111] Venkatesan S (2002). Reappraisal of bombay-karnatak version of binet simon intelligence scales (1964). Indian J Clin Psychol.

[112] Venkatesan S (2002). Extension and validation of gessells drawing test of intelligence in a group of children with communication disorders. Indian J Clin Psychol.

[113] Venkatesan S (1998). Revalidation of seguin form board test for indian children. Indian J Appl Psychol.

[114] Venkatesan S, Basavarajappa Divya M (2007). Seguin form board test: Field try out on a modified procedure of test administration. Indian J Appl Psychol.

[115] Khoshali AK, Venkatesan S (2007). Play behaviors in children with mental retardation. Psychol Stud.

[116] Karimi A, Venkatesan S (2009). Development and preliminary validation of mathematics anxiety symptoms in high school students. Asian J Dev Matters.

[117] Venkatesan S (2002). Parental attitude scale on early childhood education (PASPSE).

[118] Peshawaria R, Venkatesan S (1992). Behavior Assessment Scales for Indian Children with Mental Retardation.

[119] Venkatesan S (2004). Children with developmental disabilities: A training guide for parents, teachers and caregivers.

[120] Venkatesan S (2009). Assessment of kids with special handicaps in arithmetic and reading-writing activities’ (AKSHARA) (Venkatesan, 2009).

[121] Venkatesan S (2004). Toy kit for kids with developmental disabilities: User manual.

[122] Venkatesan S (2009). Development and standardization of computer assisted software on behavioral assessment and learning activities for kids with communication disorders (BALAK-CD).

[123] Venkatesan S, Purusotham P (2008). A profile of etiological and therapeutic searches by netizen parents/caregivers of children on the autism spectrum. JAIISH.

[124] Venkatesan S (2005). Activity log of preschool children with developmental disabilities and autism spectrum disorders. Asia Pacific Dis Rehab J.

